# Muscle Evaluation in Axial Spondyloarthritis—The Evidence for Sarcopenia

**DOI:** 10.3389/fmed.2019.00219

**Published:** 2019-10-18

**Authors:** Ana Valido, Carolina Lage Crespo, Fernando M. Pimentel-Santos

**Affiliations:** ^1^Centro Hospitalar de Lisboa Norte, Hospital de Santa Maria, Serviço de Reumatologia e Doenças Ósseas Metabólicas, Lisbon, Portugal; ^2^CEDOC - Chronic Diseases Research Center, NOVA Medical School, NOVA University of Lisbon, Lisbon, Portugal; ^3^Centro Hospitalar de Lisboa Ocidental, Hospital de Egas Moniz, Lisbon, Portugal

**Keywords:** sarcopenia, muscle strength, muscle mass, physical performance, spondyloarthritis

## Abstract

Sarcopenia is a syndrome defined as a progressive and generalized skeletal muscle disorder associated with an increased likelihood of adverse outcomes such as falls, fractures, physical disability, and death. The actual definition of sarcopenia is based on a reduction in the values of three parameters: strength, muscle mass quantity or quality, and physical performance (the determinant of severity). Muscle wasting is a common feature in several chronic diseases, such as spondyloarthritis (SpA), and significantly increases patient morbidity and mortality. Although there has been huge progress in this field over recent years, the absence of a clear definition and clear diagnostic criteria of sarcopenia has resulted in inconsistent information regarding muscle-involvement in SpA. Thus, the aim of this review is to collect relevant evidence on muscular changes occurring during the disease process from the published literature, according to the recommended tools for sarcopenia evaluation proposed by the European Working Group on Sarcopenia in Older People 2 (EWGSOP2). In addition, data from histological, electromyography, and biochemical muscle analyses of SpA patients are also reviewed. Overall, a reduction in muscle strength with a systemic decrease in lean mass seems to be associated with a gait speed compromise. This information is usually fragmented, with no studies considering the three parameters together. This paper represents a call-to-action for the design of new studies in the future.

## Introduction

Sarcopenia is a term that was first used to define age-related skeletal muscle wasting. Nowadays, it is used to describe low muscle strength with the presence of low muscle mass with/without low physical performance whenever the cause is aging, the presence of chronic disease, low protein intake, or physical inactivity (1). EWGSOP2 identifies the subcategories of sarcopenia as “primary” (age-related) or “secondary” (causal factors other than or in addition to aging are evident) and as “acute” (lasted <6 months) or “chronic” (lasted more than 6 months) (1). Furthermore, the EWGSOP has reviewed a wide range of tools for measuring specific variables of muscle strength, muscle mass, and physical performance, recommending that they be used for research purposes or in clinical practice (1, 2).

Although outside of the scope of this paper, other definitions of sarcopenia-like conditions are common in the literature, such as “cachexia” and “sarcopenic obesity.” Cachexia may be defined as the loss of lean tissue mass, with a weight loss of >5% of body weight in 12 months (or less, if in the presence of chronic illness) or with a body mass index (BMI) lower than 20, plus three of the following characteristics: decreased muscle strength, fatigue, anorexia, low fat-free mass index (FFMI), increased inflammation markers [e.g., C-reactive protein (CRP) or interleukine 6 (IL-6)], anemia, and low serum albumin (3). The spectrum of body composition in these situations varies widely in different diseases and in different disease states, from a minimal weight loss related to skeletal muscle wasting to an extreme state of loss of fat and muscle in refractory cachexia. Sarcopenic obesity represents an extreme situation that combines high muscle loss with increased fat mass and normal or high BMI (4).

**Figure 1:**
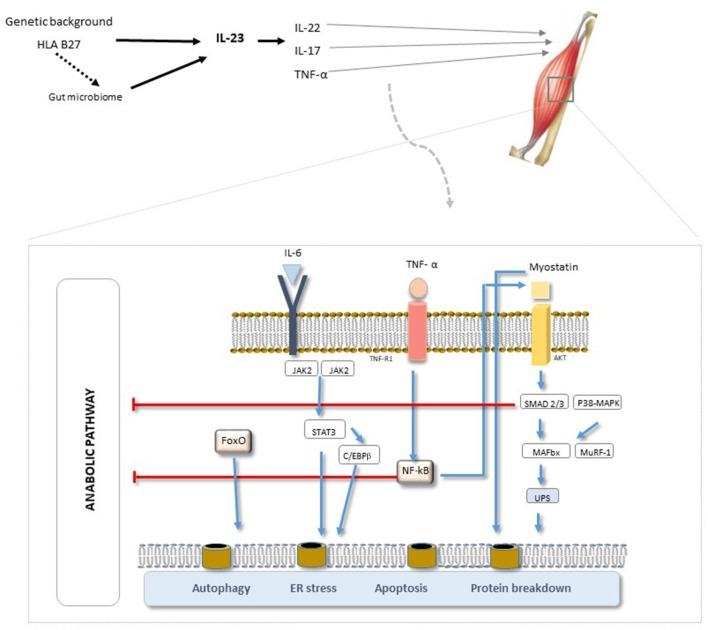
SpA pathogenesis. A complex interplay of genetic and environmental factors (biomechanics and microbiome). The triggering of the IL-17 receptor can lead to the expression of various pro-inflammatory cytokines (IL-6, IL-8, TNFα, and IL-1β). Of the various pathways involved, NF-κB has been implied to be the principal pathway and TNF-α the primary mediator involved in chronic-inflammation, inducing skeletal muscle degeneration and promoting the overall pathogenesis of sarcopenia. [Adapted from “EULAR On-Line Course on Rheumatic Diseases” and (9)]. AKT, serine/threonine protein kinase; C/EBPβ, ER, endoplasmatic reticulum; FoxO, forkhead box protein O complex; IL-6, IL-6 receptor subunit β; JAK, Janus kinase; MAFbx, muscle atrophy F-box; MuRF-1, Muscle RING Finger-1; NF-κB, factor nuclear kappa B; p38-MAPK, p38 mitogen-activated protein kinases; STAT3, signal transducer and activator of transcription 3; TNFR, TNF receptor; UPS, unfolded protein scaffold.

It has been proposed that these different concepts of muscle wasting (Table 1), i.e., sarcopenia, cachexia, and sarcopenic obesity, should be combined under the term “muscle wasting disease” (5, 6). Irrespective of the denomination, the direct consequences of this catabolic process are muscle atrophy, weakness, and physical disability combined with an increased rate of infection and premature death (7, 8). The underlying process (Figure 1) is still unknown but is likely to be a complex interplay of genetic and environmental factors (involving the microbiome and biomechanical stress (10, 11). Genetic (including HLA-B27) and intestinal microbiota changes may produce aberrant immune responses, including activation of the IL-23/-17 axis, which can lead to the expression of various pro-inflammatory cytokines (IL-6, IL-8, TNFα, and IL-1β) (7–12). It is hypothesized that the chronic inflammation driven by TNF-α induces anorexia, increases resting energy expenditure, induces muscle loss, and down-regulates anabolic hormones and growth factors (12–15). This process seems to be a common feature of several rheumatic chronic inflammatory diseases such as rheumatoid arthritis (RA) and spondyloarthritis (SpA), involving the impairment of either the contractile, metabolic, or endocrine functions of skeletal muscle (15). Further studies are necessary in this field to increase knowledge in terms of pathophysiology and, potentially, to put in evidence new therapeutic targets. In terms of prevention and therapeutic approaches for sarcopenia, the options are limited. Resistance exercise is the primary therapeutic strategy to prevent and reverse sarcopenia. In addition, leucine-enriched essential amino acid supplementation will increase muscle mass and probably function, and Vitamin D has been shown to enhance muscle function in persons with low muscle function (<50 nmol). Hormones (e.g., testosterone and Selective androgen receptor modulators) have shown some promising results, and a number of antibodies that modulate myostatin and the activin II receptor are in clinical trials (16). Another approach used to modulate the progression of sarcopenia is to counteract chronic inflammation. In this context, the administration of non-steroidal anti-inflammatory drugs, corticoids, and anti-cytokine therapy has been used with variable results (17).

**Table 1:** Definitions.

Sarcopenia	• Syndrome characterized by low muscle strength with the presence of low muscle mass with/without low physical performance.
Cachexia	• Loss of lean tissue mass, with a weight loss >5% of body weight in 12 months; Or • BMI lower than 20, plus three of the following: decreased muscle strength, fatigue, anorexia, low fat-free mass index, increase of inflammation markers such as CRP or IL-6, anemia, and low serum albumin.
Sarcopenic Obesity	• An extreme situation that combines high muscle loss with increased fat mass and normal or high BMI.

Notably, insights into muscle strength, mass, morphology, and performance in SpA patients are inconsistent and scarce. The main objective of this paper is to review and collect relevant evidence for the muscle changes occurring during the SpA process (in particular for ankylosing spondylitis [AS], the most prevalent SpA disease) based on tools for sarcopenia evaluation recommended by the EWGSOP2. In addition, data on histological, electromyography, and biochemical muscle analyses of SpA patients are also considered in the present review.

## Muscle Evaluation in SpA

### Muscle Strength

There are only a few fully validated techniques for measuring muscle strength. The handgrip strength test, requiring a calibrated handheld dynamometer, is the most highly recommended by EWGSOP2 for use in clinical practice (1). If grip measurement is not possible due to hand disability, the sit-to-stand test can be used as a proxy for the strength of quadriceps muscles (1). Previously, for research purposes, the indicated tests were the handgrip strength, knee flexion/extension, and peak expiratory flow (PEF) (2). The use of the knee flexion/extension test in clinical practice is still limited by the need for special equipment and specialized training. The PEF test cannot be recommended as an isolated procedure (2).

#### Handgrip Strength

A handgrip strength test using a handheld dynamometer is considered a reliable surrogate marker for more complicated measures of arm and leg muscle strength (18). Dynamometers are easily available and easy to use, and the method is considered to be low cost (2). Its use in clinical practice and in research studies is widespread. However, in the SpA context, there have only been a few studies where muscle strength was measured using this tool. A reduction in handgrip strength was documented in AS patients in a study by Carter et al. (19) but not in one by Marcora et al. (20). However, in the latter study, both knee extensor strength and all of the functional strength test scores (sit-to-stand test and arm curl test) were significantly lower in AS patients.

In patients with long-standing disease and radiological changes, the handgrip strength test correlates positively with aerobic power (19), and a general reduction in muscle strength has been found to be significantly associated with muscle wasting (20).

#### Sit-to-stand

When the measurement of grip is not possible due to hand disability (e.g., with advanced arthritis or stroke), the chair stand test (also called the sit-to-stand test) can be used as a proxy for the strength of leg muscles. This test measures not only strength but also endurance (1, 21).

The sit-to-stand test scores of patients with AS have been found to be significantly worse than those of healthy control subjects (20, 22). No differences have been registered between patients with different severities according to the BASMI score (22). The sit-to-stand score has been found to be significantly correlated with appendicular lean mass (20).

#### Other Tools for Muscle-Strength Evaluation

##### Knee flexion/extension

Knee flexion/extension tests are suitable for research purposes, but their use in clinical practice is restricted due to the need for special equipment (the isokinetic dynamometers that allow measurements of concentric muscle strength at various angular velocities) that is expensive and requires training of personnel (23).

Muscular strength measured by knee flexion/extension is significantly lower in AS patients than in controls (24, 25). The reduction of muscle strength seems to be related with histology (central migration of nuclei and fiber atrophy in muscle biopsies) and electromyography (lower mean power frequency) changes (26). Interestingly, it has recently been shown that the strength and endurance of lower-extremity muscles can be affected in AS patients even in the absence of peripheral joint involvement (25). Inactivity of the muscles due to inflammation, pain, stiffness, and enthesitis has been appointed as the main possible cause of this weakness and fatigue (25). Unfortunately, the molecular mechanisms underlying these interconnected processes remain obscure and warrant further investigation.

##### Peak expiratory flow

PEF is a cheap, simple, and widely accessible technique that is influenced by gender, age, physical activity, and smoking habitus (27). In individuals without lung disorders, PEF is determined by the strength of respiratory muscles. However, in some SpA patients, thoracic spine involvement and enthesitis at the costosternal, manubriosternal, costovertebral, and costotransverse joints can cause chest pain and mild to moderate reduction of chest expansion. The limitation imposed to chest expansion seems to be associated with typical restrictive defects in terms of respiratory function (28, 29) and may promote the atrophy of respiratory muscles, which may explain the reduction of respiratory muscle strength. In turn, this restrictive pattern with implicit PEF reduction has been associated with lower aerobic capacity, which depends not only on lung function but also on cardiovascular and skeletal muscle systems (19, 29). In this context, PEF data should be interpreted cautiously. Conversely, in AS patients already receiving biological therapy, the addition of targeted physiotherapy contributes to an improvement in functional parameters like PEF (30).

Overall, muscle strength evaluation, regardless of the tool selected, shows a general reduction in SpA patients. Inactivity of the muscles due to inflammation, pain, stiffness, and enthesitis is appointed as the main possible cause of this weakness (Table 2).

**Table 2:** Muscle strength in SpA.

**Muscle strength**	
Handgrip strength	• Good simple measure of muscle strength and correlates with leg strength; • Reduction in handgrip strength documented in AS patients.
Sit to Stand	• Used as a proxy for the strength of leg muscles; • Scores for patients with AS found to be significantly worse than for healthy control subjects.
Knee flexion/extension	• Limited in clinical practice by the need for special equipment and training; • Lower values of strength in AS patients than in controls; • Inactivity of the muscles related to inflammation, pain, stiffness, and enthesitis has been appointed as the main possible cause of weakness.
PEF	• Cheap, simple, and widely accessible; • Cannot be recommended as an isolated measure; • Strength of respiratory muscles may be influenced by enthesopathic inflammatory activity, deconditioning, or pain inhibition.

### Muscle Mass

Computational Tomography (CT) and Magnetic Resonance Imaging (MRI) are considered gold-standard methodologies for estimating muscle mass. However, these tools are only recommended for research purposes because of their high costs, their lack of portability, the need for highly-trained personnel to operate the equipment, and the absence of cut-off points for low muscle mass (1). On the other hand, Dual-energy X-ray absorptiometry (DXA) and Bioelectrical Impedance Analysis (BIA) are the preferred methods for use in clinical practice. These tools are affordable and widely available but are both influenced by the hydration status of the patient (2). Irrespective of technique, as muscle mass is correlated with body size, body, or appendicular skeletal muscle mass should be adjusted for body size using height squared, weight, or body mass index (31).

#### Bioelectrical Impedance Analysis (BIA)

BIA is a useful technique for assessing body composition both in healthy individuals and in patients with chronic conditions that do not show major disturbances of their water distribution. This method has seen widespread use due to its simplicity and low cost. Importantly, the results obtained with BIA strongly correlate with the data obtained with DXA (32).

There is a limited number of studies evaluating the body composition of AS patients using BIA. The only report found in the literature suggested comparable values of total body water (TBW) and fat-free mass index (FFMI) between AS patients and controls (33). However, a lower percentage of fat was highlighted in AS patients with respect to controls, though these differences were only statistically significant for males (33). In the same study, AS patients also showed a significant increase of inflammatory parameters [e.g., erythrocyte sedimentation rate (ESR) and CRP], but relevant information regarding the disease activity itself or the disease severity was not discussed. Combined results suggest that gender differences in AS patients impose dissimilarities in body composition, which has been confirmed using other tools (see below).

#### Dual-Energy X-Ray Absorptiometry (DXA)

DXA is a simple, non-invasive method using virtually no radiation that has a range of clinical applications in this field. First, it allows the assessment of associations between fat mass (FM) or lean mass (LM) and the risk of disease susceptibility. Secondly, it helps with understanding complex pathophysiological processes. Finally, DXA allows the detection of the effects of therapeutic interventions in both clinical and pre-clinical settings (34, 35).

Outcome results can be directly translated into clinical models made up of FM, non-bone LM, and bone mineral content. Such compartmentalization can be achieved in the entire body by a whole-body scan or in specific regions and has different reference values depending on gender, age, and ethnicity (34–36). This method can be used for research purposes across all age and gender groups except in pregnant women. The advantages of DXA compared with other methods such as CT or MRI are the lower radiation dose and the lower costs (34, 35). It is also far easier to perform, but it is time-consuming and hence unsuitable for large epidemiological studies (37). Moreover, different DXA instrument brands do not give consistent results (38, 39).

DXA studies in AS patients with a short disease duration (8–11 years) and mild structural changes at the rachis (≤1 syndesmophyte) have shown a non-significant reduction on the LM compared to controls (±3 kg; 4.5–5.8%) (40, 41). On the other hand, a significant reduction in LM (±6 kg; 12%) has been reported in long-standing AS patients (mean disease duration of 19 years) with severe radiological changes (≥1 syndesmophytes in 84% of the patients) (20). These studies contributed to the hypothesis of muscle wasting in AS patients. For instance, a decrease in LM has been correlated with disease activity levels measured according to the Bath Ankylosing Spondylitis Disease Activity Index (BASDAI) or the Ankylosing Spondylitis Disease Activity Score (ASDAS) (4, 15). Together with disease duration, severity and activity, gender differences may also influence the process of muscle wasting. In fact, women with AS have higher volumes of body fat than men affected by the disease, but values are still lower in comparison to the median of the reference population (15).

Considering the potential beneficial effect of TNF-α in the disease process (7, 13), it could be expected that TNF inhibitors (TNFi) would improve or prevent muscle wasting in AS. However, there are only a few studies addressing this issue, and the available data are not yet conclusive. Mounach and colleagues have not found significant differences in muscle mass, strength, or performance in AS patients treated or non-treated with TNFi. Interestingly, patients taking TNFi have significantly higher FM and fat mass index (FMI) values (4). A 2-year prospective study in TNFi-treated SpA patients documented an increase in body weight at 1 year and 2 years of treatment, mostly due to a gain in FM but also due to a significant increase in LM and in bone mineral density (BMD) (42). These results open new avenues for future research in SpA patients aiming to clarify the effect of anti-inflammatory treatments like TNFi or other therapeutics on muscle and body composition.

#### Computed Tomography (CT)

CT is a highly precise whole-body imaging system that can separate fat from the other soft tissues of the body. It is also a gold-standard method for estimating muscle mass in the research setting. However, the high costs involved, restrictions in access to equipment, and concerns about radiation exposure limit the use of this imaging method for routine clinical practice (2). It is likely that these constraints, together with a preference for MRI, are the main reasons for the scarcity of studies using CT to estimate muscle mass in SpA patients. One such study has shown a significant atrophy of the erector spinae and of multifidus muscles in patients with total bony ankylosis of the spine, but no muscle atrophy has been detected in SpA patients with mild structural changes to the spine (isolated syndesmophyte formation, vertebral squaring alone, or sacroiliac joint ankylosis with normal spinal radiographs). As expected, muscle wasting in SpA patients correlated positively with disease duration and negatively with spinal mobility (43). This is in agreement with the general results obtained using DXA. Curiously, a comparison between AS patients and patients with severe chronic mechanical back pain (CBP) revealed similar results (44). In both disease groups, gross and bilateral paraspinal muscle atrophy with fat replacement was observed in patients with a rigid spine, whereas in those with a mobile spine, muscle appearance was close to normal (44). These results suggest that the main determinant for muscle wasting is functional impairment, accompanied by limited spine movement, regardless of the inflammatory status of the patient.

#### Magnetic Resonance Imaging (MRI)

MRI is a recommended method for evaluating and monitoring changes over time in whole and regional body composition, making this method a gold standard for estimating muscle mass for research purposes (1, 2). However, high costs, limited accessibility, and concerns about radiation exposure hamper the use of this technique in clinical screening (32).

In contrast to the scarcity of studies using CT, there are relevant studies using MRI to evaluate muscle mass in the SpA context. To analyze the influence of disease duration and severity, a comparison study has been conducted in AS and in non-radiographic Axial Spondyloarthritis (nr-axSpA) patients. The cross-sectional areas (CSA) of the lumbar paravertebral muscles, including the right and left multifidus (MF), the erector spina (ES), and the psoas (PS), at multiple levels were similar between the two groups (45). However, patients with AS showed higher grades of fat infiltration than those with nr-axSpA, with this difference remaining significant after the two groups were matched for age and symptom duration. These results suggest that the paravertebral muscles in patients with SpA at different disease stages may not necessarily change in size, although their structure may differentiate toward fatty degeneration when the disease is established (45). This is an important aspect that complements the information obtained with DXA and CT. This comparison study opens the possibility that the restricted spinal movement induced by the disease is one of the main factors leading to the disuse and consequent atrophy of paravertebral muscles, with considerable impact on the patients' quality of life (45).

A current knowledge cornerstone is to understand the possible causal relationship between muscle degeneration and disease progression in SpA. To gather evidence supporting such an association in AS, a retrospective cross-sectional comparative study between AS (early and late radiologic stages) and CBP patients was conducted (46). AS patients without spine deformity (at an early stage in the radiologic course) already present a lower volume of the paraspinal muscle when compared with CBP patients. All MRI parameters for paraspinal muscle volume in early-stage AS patients are lower than in CBP patients and higher than those observed in the late stages of the disease. These data suggest that such a decrease in paraspinal muscle volume is associated with kyphotic deformity, supporting, to some extent, a causal relationship between muscle degeneration and kyphotic deformity in AS patients. Nonetheless, further study is required to prove this direct association (46). Furthermore, in contradiction of Cooper at al., where no associations were found between histological abnormalities and inflammatory cells infiltration in patients with AS (44), a recent study has shown that the inflammatory process may further influence muscle wasting (46). Also, additional data are required to clarify the mechanisms underlying the physiopathological process in AS/SpA.

Another interesting question to address in this field is whether the muscle-wasting process is limited to axial muscles or assumes a more systemic involvement with the compromise of peripheral muscles. Evidence reported by Røren Nordén and colleagues has shown that SpA is associated with reduced appendicular LM at the Quadriceps Femoris (QF) level (47). Even though no significant differences were seen in the mean QF cross-sectional area (CSA) of patients and controls, there was a trend toward lower maximal QF CSA in patients (47). As the data are not yet sufficient for muscle wasting to be considered a systemic process, the collection of additional information is of utmost relevance.

Overall, muscle mass evaluation using different recommended tools shows that there is a trend toward a general LM decrease in SpA patients involving axial and possibly peripheral muscles. Muscle wasting seems to occur in parallel with fat infiltration. Disease duration and structural deformity appear to be major determinants of muscle wasting, while inflammation might have, though this is not yet confirmed, an additional role. The effect of anti-inflammatory therapy in the process, in particular TNFi, needs clarification (Table 3).

**Table 3:** Muscle mass in SpA.

**Muscle mass tools**	
BIA	• The preferred method for use in clinical practice; simple, low-cost, and good correlation with DXA; • Gender differences impose dissimilarities in body composition in AS patients.
DXA	• The preferred alternative method for estimating muscle mass and for use in clinical practice; • Simple, noninvasive, no-radiation, lower costs; used in all age and gender groups except in pregnant women; • A significant reduction in the lean mass found in long-standing AS patients with severe radiological changes; • Muscle wasting in AS patients seems to be related with BASDAI or ASDAS.
CT	• Few studies because of high cost, limited access, radiation exposure, and preference for MRI; • Significant and positive correlations of CT score of paravertebral muscle wasting with disease duration and with restriction of spinal mobility have been established; similar results obtained when AS and severe chronic mechanical back pain patients were compared; • The main determinant for muscle wasting seems to be functional impairment, independently of the inflammatory process.
MRI	• High cost, limited accessibility, and radiation exposure; • Paravertebral muscles of patients with AS in different stages may not necessarily change in size, although their structure may change, with differentiation toward fatty degeneration in established disease; • Disease duration and structural deformity (restricted spinal movements) seem to be the major determinants of muscle wasting and the fat infiltration process.

### Physical Performance

Physical performance, a concept that involves muscles and central and peripheral nervous function, including balance (48), can be variously measured by a wide range of tests including the usual gait speed, the Short Physical Performance Battery (SPPB), the timed get-up-and-go test (TUG), the 400-meter walk, and the stair climb power (SCP) test. These tests are all currently used in clinical practice or in research except the last, which is recommended for use only for research purposes (1, 2). In the SpA context, there have been several studies evaluating gait to assess physical performance, while studies using SPPB, TUG, the 400-meter walk, and the SCP test are still scarce.

#### Gait Speed Analysis

Gait analysis is the systematic study of human motion using the eye and the brain of the observer, augmented by instrumentation, for measuring body movement, body mechanics, and muscle activity (49). Gait speed is one of several parameters commonly used to characterize gait in general (e.g., step length, stride length, cadence, dynamic base, progression line, foot angle, hip angle, squat performance). Several approaches have been used to perform these evaluations in two-dimensional (2D) or three-dimensional (3D) planes.

Gait analysis in SpA patients has long been considered an interesting research topic, as the kyphosis caused by the disease involves a shift in the center of mass downward and forward with respect to the base. This, in turn, leads to a series of adjustments made by the individual so as not to lose their balance (50). AS gait has been referred to as “walking gingerly,” as patients walk slower and have a shorter stride length than normal subjects. Yet, the cycle time and frequency do not differ between patients and control groups (51). This type of gait has been attributed to increased rigidity of the spine (in the absence of clinically and radiologically detectable peripheral joint disease), which results in decreased spinal shock absorption and, consequently, a more cautious gait (51, 52). Moreover, reduced gait velocity together with a shorter stride length can lead to an increase in the fatigue of the subject while walking (53), a common symptom in AS patients. This pattern (a trend toward a reduction in gait velocity and stride length) was recently confirmed using three-dimensional kinematics in patients who were stabilized upon TNFi treatment (with low levels of pain, stiffness, and fatigue) (54).

Gait analysis allows the planning of specific rehabilitation interventions in parallel with other therapeutic approaches aiming to prevent patients' stiffness, to improve their balance, and to avoid muscular fatigue. This represents a suitable approach for use in clinical practice to evaluate general muscle performance.

#### Other Tools for Physical Performance Evaluation

The SPPB is a composite measure of physical performance that evaluates balance, gait, strength, and endurance. This test examines: (i) an individual's ability to stand with feet together in side-by-side, semi-tandem, and tandem positions; (ii) the time needed to walk eight feet; (iii) the time to rise from a chair and return to a seated position five times (55).

In the SpA context, this test has been used to evaluate static and dynamic balance as a contributing factor that increases the risk of falls in AS patients. The results have shown that decreased SPPB scores are associated with an increased number of falls (56).

The TUG was developed for frail elderly adults aged 60–90 years referred to a geriatric hospital and targets community-dwelling frail elders. It has also been tested in other conditions including arthritis, stroke, and vertigo (57). Moreover, the TUG test has been used to investigate the effects of the balance and postural stability exercises in SpA-based rehabilitation programs (58) and the effectiveness of a progressive muscle-strengthening program using a Swiss ball in AS subjects (59). Both studies have shown an increase in the TUG score as an indicator of performance-improvement after program completion (58, 59).

The SCP test measures functional strength, balance, and agility through ascending and descending a set number of steps (60). Studies using this score have not yet been performed in SpA patients.

Overall, data evaluating muscle performance using the recommended tools are still scarce for SpA patients. Gait speed seems to be a reliable approach. Gait speed reduction and a gingerly gait put in evidence the low muscular performance and weakness of AS patients. Studies using stratified populations of patients are needed in the near future to clarify these observations (Table 4).

**Table 4:** Physical performance in SpA.

**Physical performance tools**	
Gait analysis	AS gait pattern is referred to as “walking gingerly,” as patients walk slower and have a shorter stride length than normal subjects; Has been attributed to increased rigidity of the spine, which results in decreased spinal shock absorption and consequently a more cautious gait; Determines an increase in the fatigue of the subject while walking.
SPPB	Decreased SPPB scores have been associated with an increased number of falls.
TUG	Has been used to investigate the effects on balance, postural stability, and muscle-strength of different SpA-based rehabilitation programs.
SCP, 400-meter walk	Have not been yet performed on SpA patients.

### Alternative Methods for Muscle Characterization

To complement the information obtained from muscle strength, muscle mass, and physical performance analysis, alternative methods such as histology, electromyography, and biochemical parameters were considered in this review. A summary analysis of the information available in the literature is performed in this section.

#### Histology

Muscle biopsies of AS patients reveal histological changes typically seen in muscle atrophy cases such as central migration of nuclei and reduced size of type-I and type-II muscle fibers. The distribution of muscle fibers slightly favors type-I fibers, with 4:1 mean ratios of type-I:type-II fibers. Findings of atrophy in type-II paraspinal muscle fibers provide indirect evidence that motor activation may be reduced in AS patients simply because of disuse, which is likely due to pain on movement or to reflex inhibition (26). Using specific oxidative stains of type-I and type-II fibers gives rise to a characteristic “chequerboard” pattern for both types of fibers in AS patients (26, 61). In addition, localized muscle cell damage, which reacts to acid phosphatase, correlates with the deposition of the age-related pigment lipofuscin in biopsies of AS patients. Fluorescence tests for immunoglobulins are negative in all specimens, and the modified Gomori trichrome stain does not reveal any evidence of mitochondrial damage contributing to the disease state (26, 61).

In parallel with fiber atrophy, there is histological evidence of fat infiltration with different grades of severity (44, 61). Of particular interest, an excess of perifiber and periseptal connective tissue has occasionally been reported (44, 62). The degree of fibrosis varies according to the severity of spinal disease, being highest in patients with more rigid spines but also present in patients with fully mobile spines. This assumes particular relevance for distinguishing patients with CBP from patients with AS. In fact, CBP patients with the same or a higher degree of immobility and functional impairment as AS patients show the same pattern of fiber atrophy and fat infiltration but striking differences as regards fibrosis. This suggests that paraspinal muscle fibrosis in AS represents a specific pathological process that may be related with inflammation (44) in addition to pain or reduced spinal mobility (45). However, several studies have consistently shown that histological paraspinal muscle abnormalities in AS occur without obvious inflammatory cell infiltrates (26, 44). Hence, the cause of muscle abnormalities in AS is still unknown and represents an open and interesting topic requiring future research.

#### Electromyography (EMG)

EMG studies yield variable results in patients with SpA (62). In several studies, the results were normal or revealed polyphasic motor units with slowing of all distal sensory latencies, suggesting the presence of neuropathic changes (62). Also, subclinical neurologic complications have been reported to be more frequent than clinical complications, with up to 50% of patients having myopathic features in one EMG study (45). The use of standardized methodologies in stratified populations seems to be essential for obtaining data that allow a correct interpretation of findings and a deep understanding of the muscle-wasting process. Nowadays, the electrophysiological changes and their role in disease progression remain a matter of debate.

### Biochemistry

There is still uncertainty concerning a potential role for skeletal muscle in disease susceptibility and progression. It is hypothesized that the skeletal muscle can be directly involved in the inflammatory process of AS, but the absence of inflammatory cells in muscle biopsies (26, 44) is an argument against such a hypothesis. Furthermore, muscle changes may also be a consequence of disuse due to pain associated with joint inflammation (63) or enthesitis. Serum levels of proteins of muscle origin, such as intracellular sarcoplasmic enzymes [e.g., creatine kinase (CK), aldolase, alanine aminotransferase, and aspartate aminotransferase], may be an important complement to general muscle evaluation even if conflicting data have been published. In earlier studies, raised (26, 64) or normal (65) values of CK were reported in SpA patients; on the contrary, recent studies have reported decreased values of CK in these patients (63). The discrepancies are probably due to differences in methodology between studies, and/or lacking (26, 66, 67) or inappropriate (64, 65, 68) controls. As decreasing circulating levels of several muscle enzymes (e.g., CK, Aldolase, and Creatinine) have been found to be negatively correlated with CRP levels, it has been hypothesized that muscle wasting in AS may be a consequence of disease activity (inflammation) and not only of functional restriction. In addition, the evidence that inflammation enhances the ubiquitin-proteasome pathway, which is part of an intracellular proteolytic system that contributes to the degradation of muscle proteins, seems to reinforce that hypothesis (69).

Overall, these approaches contribute to the understanding of muscle wasting, showing signals of muscle atrophy with fat and fibrosis infiltration. Certain EMG changes might contribute to this process; the role of inflammation in this process needs additional clarification (Table 5).

**Table 5:** Histology, electromiography, and biochemistry in SpA.

**Others**	
Histology	• Central migration of nuclei and reduced fiber size (type I and type II) with some atrophy; fat infiltration with different severity states; • An excess of perifiber and periseptal connective tissue occasionally shown; the degree of fibrosis varies according to the severity of spinal disease; • Fibrosis in AS might represent a specific pathological process related to muscle inflammation in addition to pain and spinal limitations.
EMG	• Yields variable results in patients with AS.
Biochemistry	• Decreasing circulating levels of several muscle enzymes have been shown to be negatively correlated with CRP levels, suggesting that muscle wasting in AS is a consequence of disease activity.

## Discussion/Conclusion

Muscular involvement may have prognostic significance in patients with AS/SpA. Several approaches have supported muscle strength reduction and muscle mass reduction, in association with physical performance compromise, favoring the diagnosis of sarcopenia, from the early stages of the disease. However, data on strength, mass, and physical performance have not been evaluated in the same group of patients simultaneously and are used independently as proxies for sarcopenia. This paper is a call-to-action for an improvement of study designs in future research to allow a clarification of the diagnosis and underlying mechanisms of sarcopenia in this context. New studies are also needed for the identification of specific biomarkers and new therapeutic targets. However, an early and targeted approach involving medical and non-medical management could be recommended in the hope of slowing down disease progression and hence spinal deformity.

## Author Contributions

AV contributed to bibliographic review and drafting of the manuscript, critically reviewed the article, and approved the final manuscript. CC critically reviewed the article and approved the final manuscript. FP-S contributed to paper conception, critically reviewed the article, and approved the final manuscript.

### Conflict of Interest

The authors declare that the research was conducted in the absence of any commercial or financial relationships that could be construed as a potential conflict of interest.
